# Supplementary material for: COVID-19 testing, timeliness and positivity from ICMR’s laboratory surveillance network in India: Profile of 176 million individuals tested and 188 million tests, March 2020 to January 2021
Source: PLoS One. 2021 Dec 3;16(12):e0260979. doi: 10.1371/journal.pone.0260979 (PMC8641892; doi:10.1371/journal.pone.0260979)
Supplement: S1 Table — (DOCX) [file pone.0260979.s001.docx]

# **S1 Table. Testing, incidence and positivity for COVID-19 among persons by states, gender and age in India (March 2020 to January 2021)**

| **States** | **Persons**  **tested**  **(in ‘000s)** | **Persons tested per 100,000 population** | | | | | | | **Persons positive (Incidence) per 100 population** | | | | | | |
| --- | --- | --- | --- | --- | --- | --- | --- | --- | --- | --- | --- | --- | --- | --- | --- |
|  |  | **Total** | **Gender** | | **Age group** | | | | **Total** | **Gender** | | **Age group** | | | |
|  |  |  | **Male** | **Female** | **0-17** | **18-40** | **41-60** | **>60** |  | **Male** | **Female** | **0-17** | **18-40** | **41-60** | **>60** |
| Andaman and Nicobar Islands | 118.2 | 29246 | 38441.1 | 18721 | 9256.4 | 40,082.9 | 37,173.4 | 20387.5 | 1.2 | 1.6 | 0.7 | 0.2 | 1.5 | 1.9 | 1.5 |
| Andhra Pradesh | 10197.6 | 18799.8 | 20100.6 | 17480.1 | 12100.4 | 22,097.9 | 19,693.0 | 26011 | 1.7 | 2.1 | 1.4 | 0.5 | 2.1 | 2.7 | 2.6 |
| Arunachal Pradesh | 160.2 | 9402.3 | 13114 | 5439.2 | 1839.3 | 15,162.9 | 17,645.2 | 3745.9 | 0.7 | 0.9 | 0.4 | 0.1 | 0.9 | 1.5 | 0.4 |
| Assam | 4706.7 | 13088.4 | 16250.7 | 9765.4 | 3491.1 | 19,883.2 | 20,375.6 | 10310.3 | 0.6 | 0.8 | 0.4 | 0.1 | 0.8 | 1.1 | 0.8 |
| Bihar | 16515.9 | 12939.3 | 14270.1 | 11489.5 | 7767.4 | 17,883.1 | 18,513.1 | 11656.3 | 0.2 | 0.3 | 0.1 | 0 | 0.3 | 0.4 | 0.3 |
| Chandigarh | 201.5 | 16550.7 | 16871.7 | 16155.7 | 4774.1 | 21,338.8 | 21,684.4 | 25657.5 | 1.9 | 2.1 | 1.7 | 0.5 | 2.1 | 3.0 | 4.7 |
| Chhattisgarh | 3719.4 | 12119.2 | 13928.3 | 10285.4 | 3423.7 | 18,599.1 | 17,274.2 | 11743.2 | 1 | 1.3 | 0.7 | 0.2 | 1.4 | 1.8 | 1.4 |
| Dadra and Nagar Haveli | 73.2 | 14277.6 | 19378 | 7676.3 | 2958.2 | 19,166.3 | 29,713.6 | 9279.4 | 0.3 | 0.4 | 0.2 | 0.1 | 0.4 | 0.9 | 0.6 |
| Daman and Diu | 39.9 | 11141.2 | 12024 | 9699.6 | 3876.1 | 12,194.2 | 22,130.8 | 11168.6 | 0.5 | 0.6 | 0.4 | 0.1 | 0.5 | 1.2 | 1 |
| Delhi | 9503.3 | 47610 | 55468 | 38516.1 | 15464.6 | 63,685.0 | 65,799.3 | 55096.9 | 3.3 | 3.7 | 2.7 | 0.7 | 3.7 | 5.4 | 8.1 |
| Goa | 441.6 | 28195.9 | 36503.9 | 19646 | 9717.3 | 39,862.0 | 30,215.2 | 24656.9 | 3.7 | 4.5 | 2.8 | 1.5 | 4.3 | 4.6 | 4.6 |
| Gujarat | 8562.6 | 12088.6 | 15042.6 | 8872.3 | 2270.7 | 18,095.9 | 16,145.5 | 16624.5 | 0.4 | 0.6 | 0.3 | 0 | 0.6 | 0.7 | 1.4 |
| Haryana | 4644.3 | 15559.6 | 17935 | 12836 | 7444.9 | 20,787.6 | 21,117.7 | 14449.9 | 1 | 1.2 | 0.8 | 0.2 | 1.2 | 1.8 | 1.8 |
| Himachal Pradesh | 878.6 | 11471.4 | 14822 | 8009.2 | 2257.6 | 17,626.1 | 14,460.6 | 10238.1 | 0.7 | 0.9 | 0.5 | 0.1 | 1.0 | 1.1 | 1 |
| Jammu and Kashmir | 2266.4 | 15264.1 | 20629.6 | 9244.1 | 3379 | 24,260.3 | 24,634.8 | 13112 | 0.9 | 1.2 | 0.6 | 0.2 | 1.2 | 1.8 | 1.7 |
| Jharkhand | 4551.8 | 11501.3 | 13443.4 | 9443.2 | 4401.6 | 17,646.3 | 17,355.9 | 9014.2 | 0.3 | 0.4 | 0.2 | 0.1 | 0.5 | 0.6 | 0.5 |
| Karnataka | 15032.5 | 21595.4 | 24700.2 | 18376.7 | 8571.5 | 29,188.3 | 27,185.1 | 20590.7 | 1.4 | 1.7 | 1.1 | 0.3 | 1.6 | 2.1 | 2.4 |
| Kerala | 7515.1 | 21545.6 | 26049.6 | 17391.3 | 7090.8 | 33,061.2 | 20,373.9 | 23825.1 | 2.1 | 2.5 | 1.7 | 1 | 2.7 | 2.3 | 2.6 |
| Ladakh | 82.9 | 24974.6 | 31523.9 | 16229.8 | 6329.5 | 33,203.7 | 39,261.1 | 20477.7 | 2.8 | 3.3 | 2.2 | 0.9 | 3.2 | 5.2 | 3.8 |
| Lakshadweep | 3.3 | 4803.3 | 6723.6 | 2774.5 | 1694.8 | 6,902.9 | 5,491.3 | 4469.3 | 0 | 0 | 0 | 0 | - | - | 0 |
| Madhya Pradesh | 4973.5 | 5796.7 | 6404.4 | 5139.5 | 1735.7 | 8,785.0 | 8,662.2 | 6218.5 | 0.3 | 0.4 | 0.2 | 0.1 | 0.4 | 0.6 | 0.7 |
| Maharashtra | 13081.4 | 10185.7 | 12249 | 7961.7 | 2649.8 | 14,395.3 | 13,213.7 | 12406.5 | 1.6 | 1.9 | 1.3 | 0.4 | 1.8 | 2.4 | 3 |
| Manipur | 464.1 | 12674.5 | 15202.2 | 10103.1 | 3456.5 | 18,877.4 | 18,291.3 | 11010 | 0.8 | 1 | 0.6 | 0.2 | 1.1 | 1.5 | 1.3 |
| Meghalaya | 256 | 6912 | 8578.4 | 5226.5 | 1360.3 | 11,766.0 | 13,710.5 | 5629.9 | 0.4 | 0.5 | 0.3 | 0.1 | 0.6 | 1.0 | 0.5 |
| Mizoram | 182.2 | 13737.7 | 18790.8 | 8550.7 | 4051.2 | 20,864.5 | 21,502.7 | 7606.3 | 0.3 | 0.5 | 0.2 | 0.1 | 0.4 | 0.6 | 0.2 |
| Nagaland | 69.1 | 3509.1 | 4766.1 | 2157.9 | 451 | 5,719.6 | 6,736.7 | 1984.9 | 0.4 | 0.6 | 0.1 | 0 | 0.6 | 1.0 | 0.2 |
| Odisha | 7271.6 | 15391.4 | 17352.1 | 13382.8 | 6802.7 | 21,495.8 | 18,472.5 | 16032.4 | 0.8 | 1 | 0.5 | 0.2 | 1.0 | 1.2 | 1.1 |
| Puducherry | 324.1 | 20786.8 | 23068.2 | 18577.9 | 10847.3 | 26,596.2 | 21,340.4 | 25139.2 | 2.5 | 3.1 | 2 | 0.8 | 3.1 | 3.4 | 3.6 |
| Punjab | 4147.6 | 13298.1 | 17058.8 | 9082.9 | 2841.3 | 20,030.9 | 17,721.6 | 10498.1 | 0.6 | 0.8 | 0.4 | 0.1 | 0.7 | 0.9 | 1 |
| Rajasthan | 4913.2 | 6023.7 | 7990.3 | 3896.9 | 1445.4 | 9,315.5 | 9,423.8 | 8563.4 | 0.6 | 0.8 | 0.4 | 0.1 | 0.8 | 1.1 | 1.4 |
| Sikkim | 54.2 | 7952.9 | 8502.3 | 7332.4 | 1483.8 | 11,311.1 | 12,426.6 | 7729.5 | 0.8 | 0.9 | 0.6 | 0.1 | 1.0 | 1.5 | 1.1 |
| Tamil Nadu | 14607.9 | 17769.5 | 19365.4 | 16138.8 | 4894.1 | 25,383.2 | 19,546.9 | 20501 | 1 | 1.2 | 0.8 | 0.2 | 1.3 | 1.4 | 1.8 |
| Telangana | 6372.5 | 16485.8 | 18540.2 | 14406.3 | 5192.2 | 22,882.7 | 22,994.7 | 17050.9 | 0.3 | 0.3 | 0.2 | 0 | 0.3 | 0.4 | 0.5 |
| Tripura | 489.9 | 11773.2 | 16041.7 | 7322.3 | 2589.6 | 17,419.6 | 16,226.4 | 10725.4 | 0.7 | 1 | 0.5 | 0.1 | 0.9 | 1.2 | 1 |
| Uttar Pradesh | 20906.4 | 8864.6 | 10554.6 | 7011.7 | 3444.7 | 13,480.8 | 14,124.9 | 8188.6 | 0.3 | 0.3 | 0.2 | 0.1 | 0.4 | 0.5 | 0.5 |
| Uttarakhand | 2043.3 | 17346.8 | 22376.7 | 12104.6 | 5580.2 | 26,312.6 | 25,187.8 | 14000.3 | 0.8 | 1.1 | 0.6 | 0.2 | 1.1 | 1.5 | 1.2 |
| West Bengal | 7095.7 | 6917.6 | 7786.5 | 5998.6 | 2030.1 | 9,746.3 | 8,511.3 | 9193.6 | 0.6 | 0.7 | 0.4 | 0.1 | 0.7 | 0.8 | 1.5 |
| **All India** | **176467.6** | **12583.9** | **14642.1** | **10392.5** | **4549** | **18,152.4** | **16,937.9** | **13799.2** | **0.8** | **0.9** | **0.6** | **0.2** | **1.0** | **1.3** | **1.5** |
