# Supplementary material for: COVID-19 testing, timeliness and positivity from ICMR’s laboratory surveillance network in India: Profile of 176 million individuals tested and 188 million tests, March 2020 to January 2021
Source: PLoS One. 2021 Dec 3;16(12):e0260979. doi: 10.1371/journal.pone.0260979 (PMC8641892; doi:10.1371/journal.pone.0260979)
Supplement: S2 Table — (DOCX) [file pone.0260979.s002.docx]

# **S2 Table. Percentage positive for COVID-19 among persons tested by states, gender and age in India (March 2020 to January 2021)**

| **States** | **Number of testing labs** | **Total persons** | **Total persons** | **Percentage of persons positive among tested** | | | | | | |
| --- | --- | --- | --- | --- | --- | --- | --- | --- | --- | --- |
|  |  | **tested (in ‘000s)** | **positive (in ‘000s)** | **Total** | **Gender** | | **Age group** | | | |
|  |  |  |  |  | **Male** | **Female** | **0-17** | **18-40** | **41-60** | **>60** |
| Andaman and Nicobar Islands | 5 | 118.2 | 4.7 | 4 | 4.1 | 3.8 | 2.3 | 3.6 | 5.0 | 7.2 |
| Andhra Pradesh | 122 | 10197.6 | 940.3 | 9.2 | 10.3 | 8 | 3.8 | 10.9 | 12.9 | 10.1 |
| Arunachal Pradesh | 23 | 160.2 | 11.4 | 7.1 | 7.2 | 6.9 | 7.3 | 6.8 | 8.2 | 9.8 |
| Assam | 48 | 4706.7 | 217.1 | 4.6 | 5 | 4 | 3.1 | 4.2 | 5.7 | 8.1 |
| Bihar | 63 | 16515.9 | 263.2 | 1.6 | 2 | 1.1 | 0.6 | 1.7 | 2.6 | 2.7 |
| Chandigarh | 8 | 201.5 | 23.5 | 11.6 | 12.4 | 10.7 | 11.1 | 9.6 | 14.9 | 18.1 |
| Chhattisgarh | 47 | 3719.4 | 306.1 | 8.2 | 9 | 7.2 | 7.2 | 7.2 | 10.5 | 11.9 |
| Dadra and Nagar Haveli | 1 | 73.2 | 1.7 | 2.4 | 2.2 | 2.8 | 2.1 | 1.8 | 4.6 | 7 |
| Daman and Diu | 0 | 39.9 | 1.8 | 4.6 | 4.9 | 3.9 | 2.8 | 4.1 | 6.2 | 8.8 |
| Delhi | 92 | 9503.3 | 648.9 | 6.8 | 6.8 | 6.9 | 4.2 | 5.4 | 10.2 | 14.7 |
| Goa | 9 | 441.6 | 57.4 | 13 | 12.3 | 14.4 | 15 | 10.7 | 15.8 | 18.5 |
| Gujarat | 88 | 8562.6 | 315.7 | 3.7 | 3.8 | 3.5 | 1.9 | 2.5 | 5.1 | 8.4 |
| Haryana | 55 | 4644.3 | 304.8 | 6.6 | 6.9 | 6.1 | 3 | 6.1 | 9.3 | 12.2 |
| Himachal Pradesh | 32 | 878.6 | 53.8 | 6.1 | 5.9 | 6.5 | 6.6 | 5.2 | 6.9 | 9.6 |
| Jammu and Kashmir | 29 | 2266.4 | 135.9 | 6 | 5.8 | 6.6 | 5.7 | 4.9 | 7.5 | 13.1 |
| Jharkhand | 43 | 4551.8 | 126.8 | 2.8 | 3.2 | 2.1 | 1.2 | 2.6 | 4.1 | 5.8 |
| Karnataka | 176 | 15032.5 | 954.6 | 6.3 | 6.7 | 5.8 | 3.7 | 5.3 | 9.5 | 11.9 |
| Kerala | 141 | 7515.1 | 731.9 | 9.7 | 9.5 | 10.1 | 13.9 | 9.5 | 10.7 | 10.8 |
| Ladakh | 4 | 82.9 | 9.3 | 11.3 | 10.4 | 13.5 | 13.8 | 9.6 | 13.7 | 18.7 |
| Lakshadweep | 2 | 3.3 | 0 | 0.100 | 0.100 | 0.000 | 0.000 | 0.1 | 0.2 | 0.000 |
| Madhya Pradesh | 104 | 4973.5 | 276.9 | 5.6 | 6.3 | 4.6 | 3.3 | 4.3 | 8.2 | 11.6 |
| Maharashtra | 207 | 13081.4 | 2026.7 | 15.5 | 15.3 | 15.8 | 15.3 | 12.6 | 18.1 | 23.8 |
| Manipur | 14 | 464.1 | 30.7 | 6.6 | 6.8 | 6.3 | 6.9 | 5.8 | 7.9 | 11.4 |
| Meghalaya | 19 | 256 | 14.3 | 5.6 | 5.5 | 5.6 | 7.2 | 4.9 | 7.0 | 8.2 |
| Mizoram | 11 | 182.2 | 4.4 | 2.4 | 2.5 | 2.1 | 2.4 | 2.3 | 3.0 | 2.4 |
| Nagaland | 14 | 69.1 | 7.8 | 11.2 | 13.1 | 6.8 | 10.3 | 11.2 | 11.5 | 12.4 |
| Odisha | 56 | 7271.6 | 360 | 5 | 5.7 | 3.9 | 2.4 | 4.7 | 6.5 | 6.6 |
| Puducherry | 14 | 324.1 | 39.6 | 12.2 | 13.3 | 11 | 7.8 | 11.4 | 15.2 | 14.4 |
| Punjab | 57 | 4147.6 | 196.7 | 4.7 | 4.7 | 4.8 | 3.6 | 3.8 | 5.9 | 9.9 |
| Rajasthan | 66 | 4913.2 | 474.5 | 9.7 | 9.6 | 9.8 | 6.5 | 8.1 | 12.4 | 16.2 |
| Sikkim | 3 | 54.2 | 5.5 | 10.1 | 11 | 8.8 | 8.9 | 8.9 | 13.2 | 14 |
| Tamil Nadu | 247 | 14607.9 | 839.7 | 5.7 | 6.4 | 5 | 4.9 | 4.7 | 6.9 | 8.9 |
| Telangana | 90 | 6372.5 | 97.2 | 1.5 | 1.8 | 1.2 | 1 | 1.2 | 1.8 | 2.6 |
| Tripura | 5 | 489.9 | 29.8 | 6.1 | 6 | 6.3 | 5.2 | 5.4 | 7.4 | 9.1 |
| Uttar Pradesh | 229 | 20906.4 | 640.6 | 3.1 | 3.3 | 2.7 | 1.6 | 2.8 | 4.5 | 6.1 |
| Uttarakhand | 62 | 2043.3 | 95.8 | 4.7 | 4.8 | 4.6 | 3 | 4.1 | 6.5 | 8.9 |
| West Bengal | 122 | 7095.7 | 565.5 | 8 | 8.4 | 7.3 | 4.2 | 6.0 | 10.3 | 15.9 |
| **All India** | **2316** | **176467.6** | **10814.2** | **6.1** | **6.4** | **5.7** | **3.6** | **5.2** | **8.4** | **10.9** |
