# Supplementary material for: COVID-19 testing, timeliness and positivity from ICMR’s laboratory surveillance network in India: Profile of 176 million individuals tested and 188 million tests, March 2020 to January 2021
Source: PLoS One. 2021 Dec 3;16(12):e0260979. doi: 10.1371/journal.pone.0260979 (PMC8641892; doi:10.1371/journal.pone.0260979)
Supplement: S3 Table — (DOCX) [file pone.0260979.s003.docx]

# **S3 Table. Testing characteristics of the laboratory surveillance network for COVID-19 by states in India (March 2020 to January 2021)**

| **States** | **Total tests** | **Tests per 100,000 population** | **% Symptomatic among tested** | **% Positive among tested** | **% of individuals tested more than once** | **% Tests carried out by phases** | | |
| --- | --- | --- | --- | --- | --- | --- | --- | --- |
|  |  |  |  |  |  | **Phase I** | **Phase II** | **Phase III** |
| Andaman and Nicobar Islands | 126529 | 31,319.3 | 2.6 | 3.9 | 4.8 | 6.0 | 16.8 | 77.3 |
| Andhra Pradesh | 11923397 | 21,981.4 | 2.3 | 8.2 | 11.8 | 2.7 | 25.7 | 71.6 |
| Arunachal Pradesh | 170950 | 10,032.4 | 1.4 | 7.0 | 5.0 | 3.7 | 49.6 | 46.7 |
| Assam | 4995458 | 13,891.3 | 1.1 | 4.5 | 4.8 | 1.1 | 27.8 | 71.1 |
| Bihar | 17665462 | 13,840.0 | 2.9 | 1.5 | 5.2 | 0.4 | 14.7 | 84.9 |
| Chandigarh | 215328 | 17,687.4 | 9.8 | 11.2 | 5.0 | 2.7 | 15.2 | 82.1 |
| Chhattisgarh | 3820184 | 12,447.7 | 9.7 | 8.2 | 2.3 | 1.4 | 10.1 | 88.5 |
| Dadra and Nagar Haveli | 75609 | 14,752.3 | 7.1 | 2.3 | 2.9 | 11.6 | 35.7 | 52.7 |
| Daman and Diu | 41967 | 11,714.2 | 12.1 | 4.4 | 4.1 | 8.7 | 37.4 | 53.9 |
| Delhi | 10262620 | 51,414.1 | 8.7 | 6.7 | 5.4 | 1.6 | 13.5 | 84.9 |
| Goa | 468062 | 29,886.9 | 7.2 | 12.8 | 4.3 | 2.5 | 40.0 | 57.4 |
| Gujarat | 8808912 | 12,436.4 | 18.0 | 3.7 | 2.2 | 2.3 | 16.8 | 80.8 |
| Haryana | 4891861 | 16,388.8 | 11.4 | 6.8 | 3.9 | 2.6 | 20.7 | 76.7 |
| Himachal Pradesh | 917745 | 11,982.0 | 3.7 | 6.2 | 3.4 | 3.7 | 20.7 | 75.6 |
| Jammu and Kashmir | 2344841 | 15,792.2 | 2.8 | 6.2 | 2.8 | 4.0 | 26.6 | 69.5 |
| Jharkhand | 4769693 | 12,051.8 | 1.2 | 2.9 | 3.8 | 1.1 | 12.9 | 86.0 |
| Karnataka | 16097462 | 23,125.3 | 5.0 | 6.2 | 5.2 | 1.8 | 15.6 | 82.6 |
| Kerala | 8113700 | 23,261.8 | 8.3 | 9.8 | 6.2 | 0.8 | 8.3 | 90.9 |
| Ladakh | 89582 | 26,982.4 | 3.5 | 11.0 | 5.4 | 7.8 | 24.5 | 67.7 |
| Lakshadweep | 3421 | 5,022.1 | 0.9 | 0.1 | 4.0 | 40.5 | 51.9 | 7.6 |
| Madhya Pradesh | 5121664 | 5,969.3 | 15.7 | 5.7 | 2.3 | 3.1 | 23.9 | 73.0 |
| Maharashtra | 13785775 | 10,734.2 | 12.1 | 15.5 | 3.9 | 3.3 | 25.1 | 71.6 |
| Manipur | 505991 | 13,819.6 | 0.7 | 6.4 | 6.5 | 1.8 | 23.7 | 74.6 |
| Meghalaya | 277397 | 7,489.8 | 4.4 | 5.6 | 5.5 | 1.5 | 18.4 | 80.1 |
| Mizoram | 199435 | 15,034.1 | 1.2 | 2.3 | 7.1 | 0.4 | 19.6 | 80.0 |
| Nagaland | 78653 | 3,996.2 | 0.5 | 12.2 | 9.1 | 1.5 | 34.7 | 63.8 |
| Odisha | 7471769 | 15,815.0 | 6.1 | 4.9 | 2.3 | 2.0 | 21.6 | 76.4 |
| Puducherry | 339796 | 21,791.3 | 15.0 | 11.8 | 3.9 | 1.8 | 18.2 | 80.0 |
| Punjab | 4333005 | 13,892.5 | 2.7 | 4.6 | 3.5 | 1.9 | 21.7 | 76.4 |
| Rajasthan | 5167121 | 6,335.0 | 7.5 | 10.0 | 3.9 | 6.5 | 33.1 | 60.5 |
| Sikkim | 60618 | 8,901.5 | 5.4 | 10.6 | 8.2 | 3.5 | 40.5 | 56.0 |
| Tamil Nadu | 15213805 | 18,506.5 | 7.1 | 5.7 | 3.3 | 2.8 | 27.1 | 70.0 |
| Telangana | 6521503 | 16,871.2 | 24.1 | 1.5 | 1.9 | 0.5 | 14.9 | 84.7 |
| Tripura | 516648 | 12,416.1 | 8.8 | 5.8 | 4.1 | 3.3 | 43.4 | 53.3 |
| Uttar Pradesh | 23262309 | 9,863.6 | 1.6 | 2.9 | 8.6 | 1.1 | 19.3 | 79.6 |
| Uttarakhand | 2111112 | 17,922.9 | 4.7 | 4.7 | 2.6 | 1.2 | 15.7 | 83.0 |
| West Bengal | 7580592 | 7,390.4 | 8.1 | 7.9 | 4.9 | 2.1 | 21.0 | 76.9 |
| **All India** | **188349976** | **13,412.8** | **7.0** | **6.0** | **5.1** | **2.0** | **19.8** | **78.2** |

Note: * Phase I – 01-03-2020 to 31-05-2020, Phase II – 01-06-2020 to 31-08-2020, Phase III – >01-09-2020
