# Supplementary material for: COVID-19 testing, timeliness and positivity from ICMR’s laboratory surveillance network in India: Profile of 176 million individuals tested and 188 million tests, March 2020 to January 2021
Source: PLoS One. 2021 Dec 3;16(12):e0260979. doi: 10.1371/journal.pone.0260979 (PMC8641892; doi:10.1371/journal.pone.0260979)
Supplement: S5 Table — (DOCX) [file pone.0260979.s005.docx]

# **S5 Table. Timeliness of the laboratory surveillance network for COVID-19 by states and test result in India (March 2020 to January 2021)**

| **States** | **Symptom onset to sample collection** | | | | **Sample collection to testing** | | | | **Sample testing to data entry** | | | |
| --- | --- | --- | --- | --- | --- | --- | --- | --- | --- | --- | --- | --- |
|  | **Negative test** | | **Positive test** | | **Negative test** | | **Positive test** | | **Negative test** | | **Positive test** | |
|  | **Median** | **IQR** | **Median** | **IQR** | **Median** | **IQR** | **Median** | **IQR** | **Median** | **IQR** | **Median** | **IQR** |
| Andaman and Nicobar Islands | 2 | 1, 3 | 2 | 1, 3 | 0 | 0, 0 | 1 | 0, 4 | 13 | 3, 26 | 4 | 1, 21 |
| Andhra Pradesh | 0 | 0, 3 | 2 | 1, 4 | 1 | 0, 2 | 1 | 0, 2 | 0 | 0, 1 | 0 | 0, 2 |
| Arunachal Pradesh | 2 | 1, 5 | 2 | 1, 4 | 0 | 0, 1 | 0 | 0, 0 | 1 | 0, 5 | 1 | 0, 2 |
| Assam | 0 | 0, 2 | 2 | 0, 3 | 0 | 0, 1 | 0 | 0, 0 | 0 | 0, 2 | 0 | 0, 1 |
| Bihar | 2 | 0, 3 | 2 | 0, 4 | 1 | 0, 2 | 1 | 0, 2 | 0 | 0, 0 | 0 | 0, 1 |
| Chandigarh | 3 | 1, 4 | 3 | 2, 4 | 0 | 0, 0 | 0 | 0, 1 | 0 | 0, 1 | 0 | 0, 1 |
| Chhattisgarh | 2 | 0, 3 | 2 | 1, 3 | 0 | 0, 1 | 0 | 0, 1 | 0 | 0, 2 | 0 | 0, 1 |
| Dadra and Nagar Haveli | 0 | 0, 2 | 0 | 0, 1 | 0 | 0, 0 | 0 | 0, 0 | 0 | 0, 0 | 0 | 0, 0 |
| Daman and Diu | 1 | 0, 3 | 1 | 0, 3 | 1 | 0, 1 | 0 | 0, 1 | 0 | 0, 0 | 0 | 0, 0 |
| Delhi | 2 | 1, 4 | 3 | 2, 5 | 0 | 0, 0 | 0 | 0, 1 | 0 | 0, 1 | 0 | 0, 1 |
| Goa | 1 | 0, 3 | 1 | 1, 3 | 0 | 0, 1 | 0 | 0, 1 | 1 | 0, 1 | 0 | 0, 1 |
| Gujarat | 1 | 1, 3 | 2 | 1, 4 | 0 | 0, 0 | 0 | 0, 1 | 1 | 0, 9 | 0 | 0, 1 |
| Haryana | 2 | 0, 4 | 3 | 1, 4 | 1 | 0, 1 | 1 | 0, 1 | 1 | 0, 4 | 0 | 0, 1 |
| Himachal Pradesh | 2 | 0, 4 | 2 | 1, 4 | 1 | 0, 1 | 1 | 0, 1 | 0 | 0, 1 | 0 | 0, 0 |
| Jammu and Kashmir | 0 | 0, 2 | 2 | 0, 4 | 0 | 0, 1 | 1 | 0, 1 | 2 | 0, 10 | 2 | 1, 6 |
| Jharkhand | 0 | 0, 2 | 2 | 1, 3 | 0 | 0, 3 | 0 | 0, 2 | 0 | 0, 3 | 1 | 0, 2 |
| Karnataka | 1 | 0, 3 | 2 | 1, 4 | 1 | 0, 2 | 0 | 0, 1 | 0 | 0, 1 | 0 | 0, 0 |
| Kerala | 3 | 1, 5 | 3 | 2, 7 | 0 | 0, 0 | 0 | 0, 0 | 10 | 2, 22 | 5 | 1, 15 |
| Ladakh | 2 | 0, 6 | 3 | 2, 7 | 1 | 0, 2 | 1 | 1, 2 | 0 | 0, 1 | 0 | 0, 0 |
| Lakshadweep | 3.5 | 2.75, 9.5 | NA | NA, NA | 1 | 0, 1 | 1 | 0.75, 1 | 1 | 0, 3 | 0 | 0, 3.25 |
| Madhya Pradesh | 2 | 1, 4 | 2 | 1, 4 | 1 | 0, 2 | 1 | 0, 1 | 0 | 0, 1 | 0 | 0, 1 |
| Maharashtra | 2 | 0, 3 | 2 | 1, 4 | 0 | 0, 1 | 0 | 0, 1 | 0 | 0, 2 | 0 | 0, 1 |
| Manipur | 2 | 0, 4 | 2 | 1, 5 | 0 | 0, 1 | 0 | 0, 1 | 0 | 0, 2 | 0 | 0, 1 |
| Meghalaya | 2 | 1, 5 | 3 | 1, 7 | 0 | 0, 0 | 1 | 0, 1 | 1 | 0, 17 | 1 | 0, 1 |
| Mizoram | 2 | 1, 5 | 2 | 1, 5 | 1 | 0, 2 | 1 | 0, 2 | 0 | 0, 0 | 0 | 0, 0 |
| Nagaland | 2 | 1, 4 | 3 | 2, 4 | 1 | 0, 2 | 2 | 1, 2 | 4 | 1, 13 | 7 | 3, 10 |
| Odisha | 2 | 0, 4 | 3 | 2, 4 | 0 | 0, 0 | 0 | 0, 1 | 0 | 0, 0 | 0 | 0, 1 |
| Puducherry | 2 | 1, 3 | 2 | 1, 3 | 0 | 0, 1 | 0 | 0, 1 | 0 | 0, 0 | 0 | 0, 0 |
| Punjab | 3 | 1, 7 | 3 | 1, 4 | 1 | 0, 1 | 1 | 0, 1 | 0 | 0, 1 | 0 | 0, 1 |
| Rajasthan | 2 | 0, 7 | 2 | 0, 3 | 1 | 0, 1 | 1 | 0, 1 | 1 | 0, 4 | 1 | 0, 3 |
| Sikkim | 0 | 0, 0 | 1 | 0, 3 | 1 | 0, 2 | 0 | 0, 1 | 1 | 1, 2 | 1 | 1, 2.75 |
| Tamil Nadu | 1 | 0, 2 | 2 | 1, 3 | 1 | 0, 1 | 1 | 0, 1 | 1 | 0, 2 | 0 | 0, 1 |
| Telangana | 2 | 1, 4 | 2 | 2, 4 | 0 | 0, 0 | 1 | 0, 1 | 0 | 0, 0 | 2 | 1, 10 |
| Tripura | 1 | 0, 2 | 2 | 1, 3 | 0 | 0, 1 | 0 | 0, 0 | 0 | 0, 1 | 0 | 0, 1 |
| Uttar Pradesh | 2 | 1, 4 | 3 | 2, 6 | 1 | 0, 1 | 0 | 0, 1 | 0 | 0, 0 | 0 | 0, 1 |
| Uttarakhand | 2 | 1, 4 | 2 | 1, 4 | 1 | 0, 2 | 1 | 0, 2 | 1 | 0, 2 | 0 | 0, 1 |
| West Bengal | 3 | 1, 5 | 3 | 2, 6 | 0 | 0, 1 | 1 | 0, 1 | 0 | 0, 1 | 0 | 0, 1 |
| **All India** | **2** | **0, 3** | **2** | **1, 4** | **0** | **0, 1** | **0** | **0, 1** | **0** | **0, 1** | **0** | **0, 1** |
