# Supplementary material for: COVID-19 testing, timeliness and positivity from ICMR’s laboratory surveillance network in India: Profile of 176 million individuals tested and 188 million tests, March 2020 to January 2021
Source: PLoS One. 2021 Dec 3;16(12):e0260979. doi: 10.1371/journal.pone.0260979 (PMC8641892; doi:10.1371/journal.pone.0260979)
Supplement: S6 Table — (DOCX) [file pone.0260979.s006.docx]

# **S6 Table. Timeliness of the laboratory surveillance network for COVID-19 by states and scale-up phases in India (March 2020 to January 2021)**

| **States** | **Symptom onset to sample collection** | | | | | | **Sample collection to testing** | | | | | | **Sample testing to data entry** | | | | | |
| --- | --- | --- | --- | --- | --- | --- | --- | --- | --- | --- | --- | --- | --- | --- | --- | --- | --- | --- |
|  | **Phase I** | | **Phase II** | | **Phase III** | | **Phase I** | | **Phase II** | | **Phase III** | | **Phase I** | | **Phase II** | | **Phase III** | |
|  | **Median** | **IQR** | **Median** | **IQR** | **Median** | **IQR** | **Median** | **IQR** | **Median** | **IQR** | **Median** | **IQR** | **Median** | **IQR** | **Median** | **IQR** | **Median** | **IQR** |
| Andaman and Nicobar Islands | 2 | 1, 4 | 2 | 1, 2 | 2 | 0, 3 | 0 | 0, 1 | 1 | 0, 2 | 0 | 0, 0 | 1 | 1, 2 | 3 | 2, 6 | 19 | 5, 28 |
| Andhra Pradesh | 0 | 0, 0 | 1 | 0, 3 | 2 | 1, 4 | 1 | 0, 2 | 1 | 0, 3 | 1 | 0, 2 | 2 | 1, 5 | 4 | 1, 12 | 0 | 0, 0 |
| Arunachal Pradesh | 4 | 2, 8.75 | 2 | 1, 6 | 2 | 1, 4 | 1 | 1, 2 | 0 | 0, 2 | 0 | 0, 0 | 0 | 0, 1 | 1 | 0, 8 | 0 | 0, 3 |
| Assam | 3 | 2, 7 | 0 | 0, 2 | 0 | 0, 2 | 2 | 1, 2 | 0 | 0, 3 | 0 | 0, 0 | 2 | 1, 4 | 1 | 0, 3 | 0 | 0, 1 |
| Bihar | 2 | 0, 4 | 2 | 0, 4 | 2 | 0, 3 | 1 | 1, 2 | 1 | 0, 1 | 1 | 0, 2 | 0 | 0, 1 | 1 | 1, 1 | 0 | 0, 0 |
| Chandigarh | 3 | 2, 6 | 3 | 1, 4 | 3 | 2, 4 | 0 | 0, 1 | 0 | 0, 1 | 0 | 0, 0 | 0 | 0, 1 | 0 | 0, 1 | 0 | 0, 1 |
| Chhattisgarh | 4 | 2, 7 | 2 | 1, 4 | 2 | 0, 3 | 1 | 1, 2 | 1 | 1, 2 | 0 | 0, 1 | 2 | 1, 4 | 2 | 0, 6 | 0 | 0, 1 |
| Dadra and Nagar Haveli | 4.5 | 2, 12.75 | 0 | 0, 1 | 0 | 0, 2 | 0 | 0, 2 | 0 | 0, 1 | 0 | 0, 0 | 0 | 0, 1 | 0 | 0, 0 | 0 | 0, 0 |
| Daman and Diu | 122.5 | 3, 124 | 1 | 0, 3 | 1 | 0, 2 | 1 | 0, 3 | 1 | 0, 1 | 1 | 0, 1 | 0 | 0, 1 | 0 | 0, 0 | 0 | 0, 0 |
| Delhi | 4 | 2, 6 | 3 | 2, 5 | 2 | 1, 4 | 1 | 0, 1 | 0 | 0, 0 | 0 | 0, 1 | 1 | 0, 2 | 0 | 0, 1 | 0 | 0, 0 |
| Goa | 3 | 1, 6 | 1 | 0, 3 | 1 | 0, 2 | 0 | 0, 1 | 0 | 0, 1 | 0 | 0, 0 | 2 | 1, 9 | 1 | 0, 2 | 0 | 0, 1 |
| Gujarat | 3 | 2, 5 | 2 | 1, 3 | 1 | 0, 2 | 0 | 0, 1 | 0 | 0, 0 | 0 | 0, 0 | 0 | 0, 1 | 0 | 0, 4 | 1 | 0, 10 |
| Haryana | 3 | 2, 7 | 3 | 1, 4 | 2 | 0, 3 | 1 | 1, 2 | 1 | 0, 1 | 1 | 0, 1 | 1 | 0, 1 | 1 | 0, 2 | 1 | 0, 5 |
| Himachal Pradesh | 3 | 2, 6 | 2 | 1, 4 | 2 | 0, 4 | 1 | 1, 1 | 1 | 1, 1 | 0 | 0, 1 | 0 | 0, 1 | 0 | 0, 1 | 0 | 0, 1 |
| Jammu and Kashmir | 0 | 0, 3 | 0 | 0, 2 | 0 | 0, 2 | 1 | 1, 2 | 1 | 1, 2 | 0 | 0, 1 | 2 | 1, 5 | 4 | 1, 10 | 1 | 0, 10 |
| Jharkhand | 1 | 0, 2.75 | 1 | 0, 2 | 0 | 0, 2 | 3 | 2, 4 | 1 | 0, 3 | 0 | 0, 3 | 3 | 1, 5 | 2 | 1, 5 | 0 | 0, 2 |
| Karnataka | 2 | 1, 4 | 2 | 0, 3 | 2 | 0, 3 | 1 | 1, 2 | 1 | 0, 2 | 1 | 0, 2 | 0 | 0, 1 | 0 | 0, 1 | 0 | 0, 1 |
| Kerala | 3 | 2, 6 | 3 | 2, 5 | 1 | 0, 3 | 1 | 1, 1 | 1 | 0, 1 | 0 | 0, 0 | 0 | 0, 1 | 1 | 0, 7 | 11 | 2, 23 |
| Ladakh | 3.5 | 2, 5.75 | 3 | 1, 14 | 2 | 0, 4 | 3 | 2, 4 | 2 | 1, 4 | 0 | 0, 1 | 1 | 1, 2 | 0 | 0, 1 | 0 | 0, 1 |
| Lakshadweep | 3 | 3, 6 | 4 | 2, 11 | NA | NA, NA | 0 | 0, 1 | 1 | 0, 1 | 0 | 0, 1 | 1 | 0, 1 | 2.5 | 0, 4 | 0 | 0, 9 |
| Madhya Pradesh | 3 | 1, 5 | 2 | 1, 4 | 2 | 1, 4 | 1 | 1, 2 | 1 | 1, 2 | 0 | 0, 1 | 1 | 0, 2 | 1 | 0, 1 | 0 | 0, 0 |
| Maharashtra | 3 | 1, 6 | 2 | 1, 4 | 2 | 0, 3 | 1 | 0, 1 | 0 | 0, 1 | 0 | 0, 1 | 1 | 0, 2 | 0 | 0, 1 | 0 | 0, 2 |
| Manipur | 3 | 2, 6 | 2 | 0, 3 | 2 | 0, 5 | 3 | 1, 4 | 1 | 1, 4 | 0 | 0, 0 | 0 | 0, 0 | 0 | 0, 1 | 0 | 0, 4 |
| Meghalaya | 3 | 1, 5 | 3 | 1, 6 | 2 | 1, 5 | 1 | 1, 1 | 0 | 0, 1 | 0 | 0, 0 | 0 | 0, 2 | 3 | 1, 24 | 1 | 0, 11 |
| Mizoram | 2 | 0, 5.25 | 1 | 1, 2 | 3 | 1, 7 | 1 | 1, 2 | 2 | 1, 4 | 0 | 0, 2 | 0 | 0, 0 | 0 | 0, 0 | 0 | 0, 0 |
| Nagaland | 3 | 1, 5 | 3 | 1.25, 4.75 | 3 | 1, 4 | 1 | 1, 1 | 2 | 0, 4 | 1 | 0, 2 | 0 | 0, 1 | 3 | 1, 10 | 6 | 0, 14 |
| Odisha | 4 | 2, 6 | 2 | 0, 4 | 2 | 1, 4 | 2 | 1, 3 | 0 | 0, 1 | 0 | 0, 0 | 1 | 0, 3 | 0 | 0, 1 | 0 | 0, 0 |
| Puducherry | 3 | 1, 6 | 2 | 2, 4 | 1 | 0, 3 | 1 | 0, 1 | 1 | 0, 2 | 0 | 0, 0 | 0 | 0, 0 | 0 | 0, 0 | 0 | 0, 0 |
| Punjab | 4 | 2, 7 | 2 | 0, 4 | 3 | 2, 6 | 1 | 1, 2 | 1 | 1, 2 | 1 | 0, 1 | 0 | 0, 1 | 1 | 0, 1 | 0 | 0, 1 |
| Rajasthan | 1 | 0, 3 | 2 | 0, 4 | 2 | 0, 11 | 1 | 0, 1 | 1 | 1, 1 | 1 | 0, 1 | 1 | 0, 2 | 1 | 0, 3 | 1 | 0, 5 |
| Sikkim | 4 | 3, 7 | 3 | 1, 4 | 0 | 0, 0 | 1 | 1, 3 | 1 | 1, 2 | 0 | 0, 1 | 3 | 1, 5 | 1 | 1, 2 | 1 | 1, 3 |
| Tamil Nadu | 2 | 0, 4 | 1 | 0, 3 | 1 | 0, 2 | 1 | 0, 1 | 1 | 0, 1 | 1 | 0, 1 | 2 | 1, 6 | 1 | 0, 3 | 1 | 0, 1 |
| Telangana | 3 | 1, 6 | 2 | 1, 4 | 2 | 1, 3 | 1 | 0, 1 | 0 | 0, 1 | 0 | 0, 0 | 1 | 0, 7 | 1 | 0, 5 | 0 | 0, 0 |
| Tripura | 4 | 2, 7 | 0 | 0, 2 | 1 | 0, 2 | 1 | 1, 1 | 0 | 0, 1 | 0 | 0, 1 | 1 | 1, 3 | 1 | 0, 2 | 0 | 0, 0 |
| Uttar Pradesh | 2 | 1, 4 | 2 | 1, 5 | 2 | 1, 4 | 1 | 1, 2 | 1 | 0, 2 | 0 | 0, 1 | 1 | 0, 3 | 0 | 0, 1 | 0 | 0, 0 |
| Uttarakhand | 3 | 1, 5 | 2 | 1, 4 | 2 | 0, 4 | 1 | 1, 2 | 1 | 0, 3 | 1 | 0, 2 | 0 | 0, 1 | 1 | 0, 2 | 1 | 0, 2 |
| West Bengal | 3 | 2, 6 | 3 | 2, 5 | 2 | 1, 4 | 2 | 1, 3 | 1 | 0, 2 | 0 | 0, 1 | 1 | 0, 3 | 0 | 0, 1 | 0 | 0, 1 |
| **All India** | **3** | **1, 5** | **2** | **1, 4** | **2** | **0, 3** | **1** | **0, 2** | **1** | **0, 2** | **0** | **0, 1** | **1** | **0, 3** | **1** | **0, 2** | **0** | **0, 1** |

Note: * Phase I – 01-03-2020 to 31-05-2020, Phase II – 01-06-2020 to 31-08-2020, Phase III – >01-09-2020
