# Supplementary material for: COVID-19 testing, timeliness and positivity from ICMR’s laboratory surveillance network in India: Profile of 176 million individuals tested and 188 million tests, March 2020 to January 2021
Source: PLoS One. 2021 Dec 3;16(12):e0260979. doi: 10.1371/journal.pone.0260979 (PMC8641892; doi:10.1371/journal.pone.0260979)
Supplement: S2 Fig — (PDF) [file pone.0260979.s008.pdf]

I. Group I (Total positive case > 400,000)

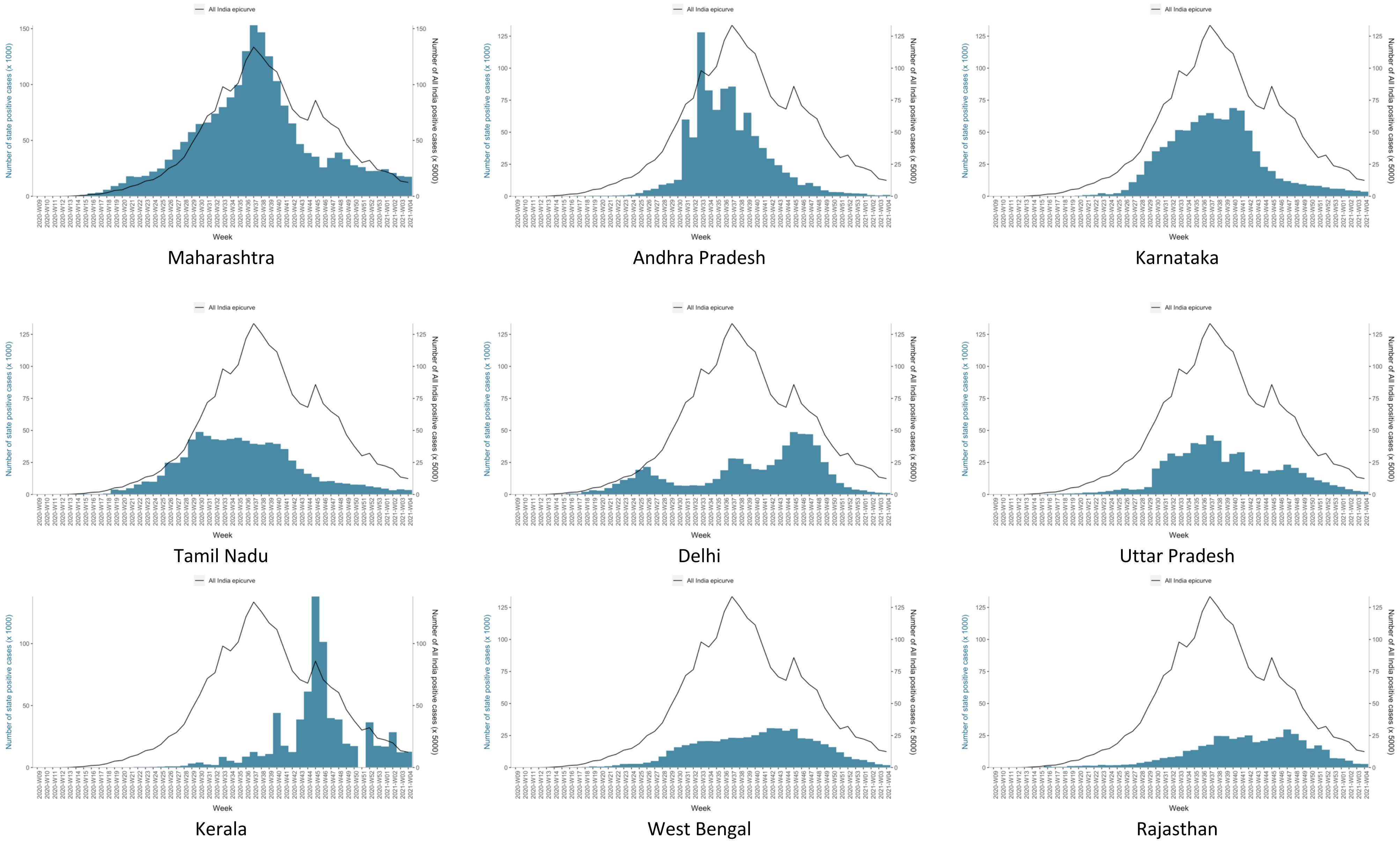

II. Group II (Total positive cases 100,000 to 400,000)

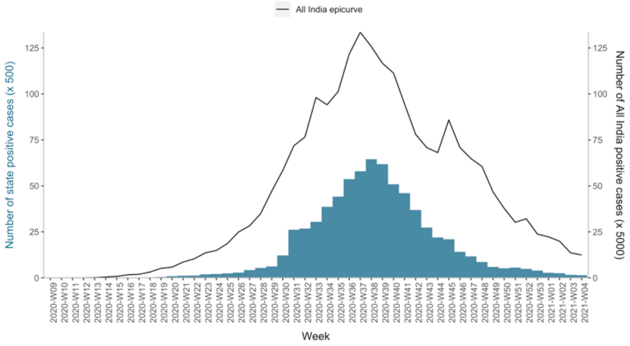

Odisha

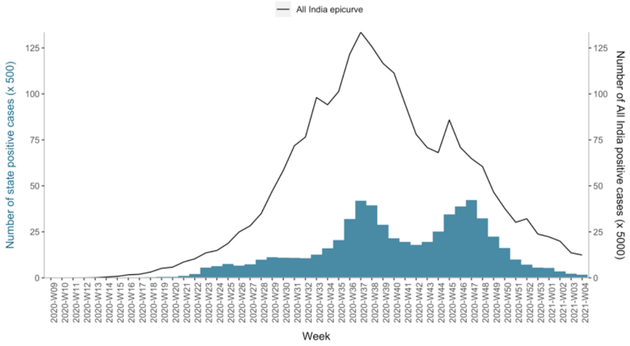

Haryana

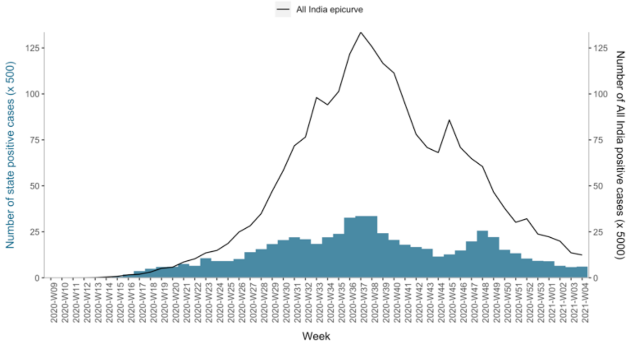

Gujarat

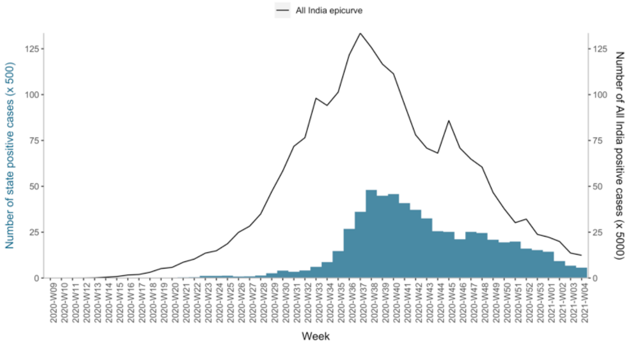

Chhattisgarh

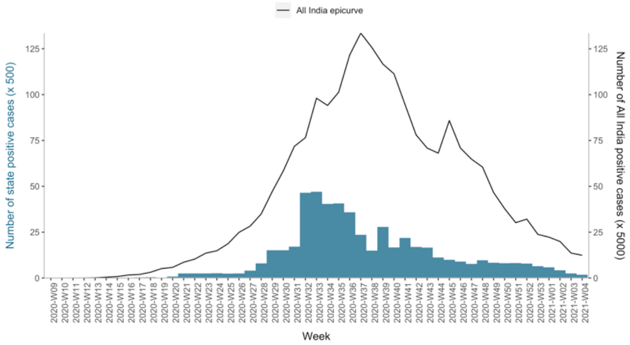

Bihar

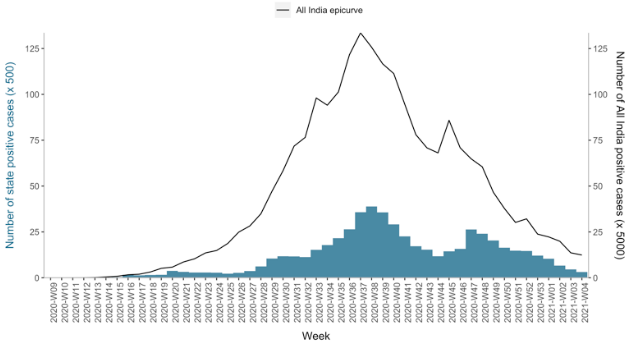

Madhya Pradesh

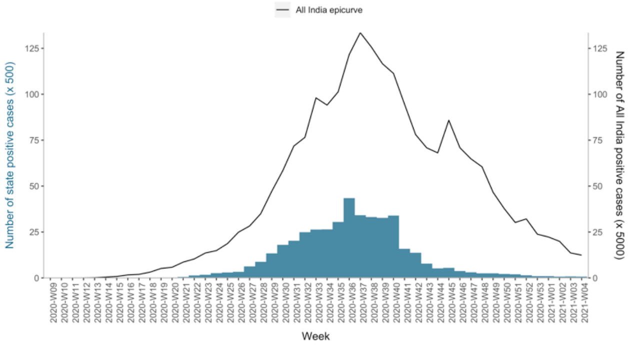

Assam

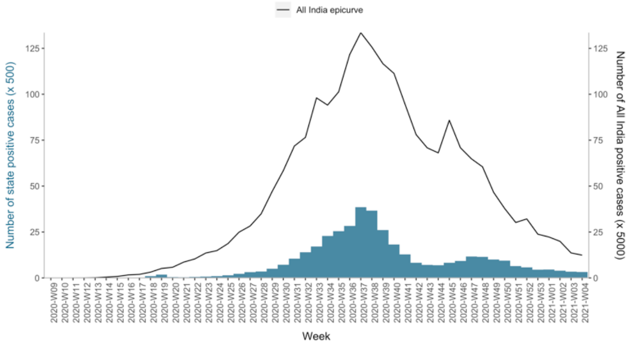

Punjab

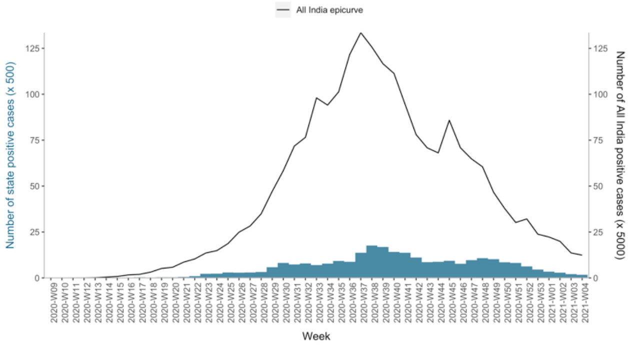

Jammu and Kashmir

III. Group III (Total positive cases <100,000)

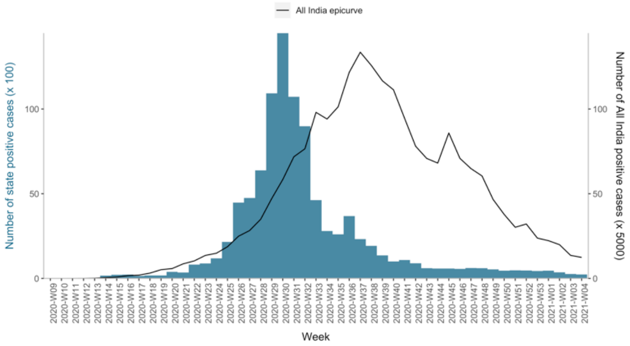

Telangana

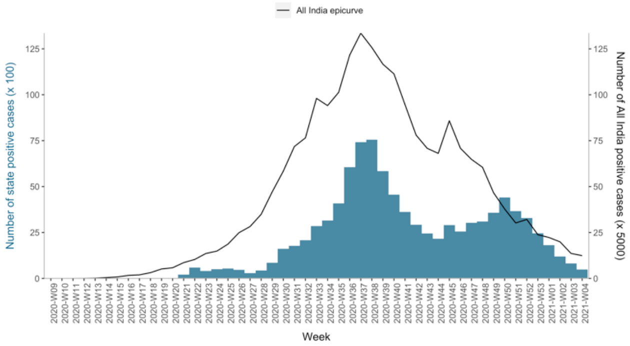

Uttarakhand

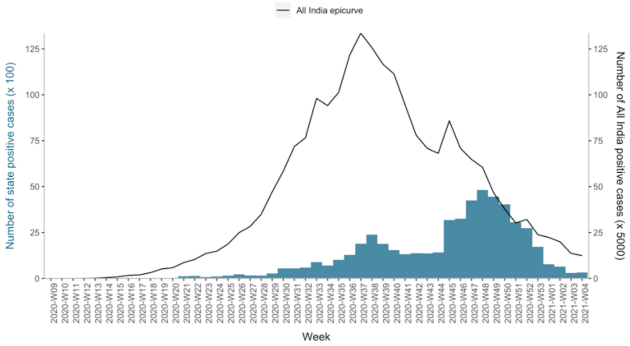

Goa

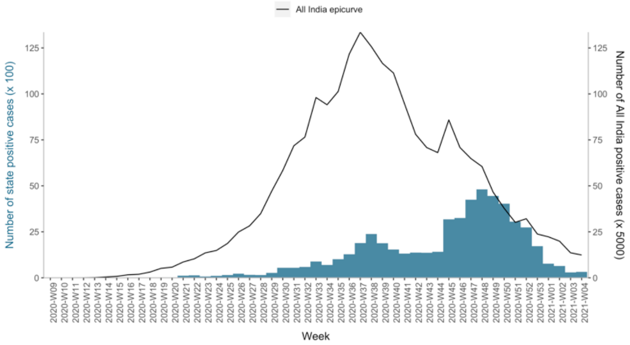

Himachal Pradesh

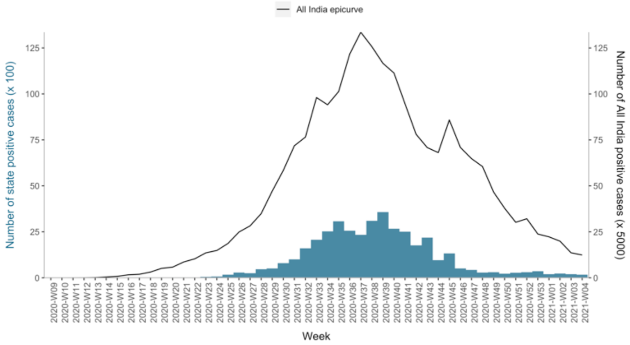

Puducherry

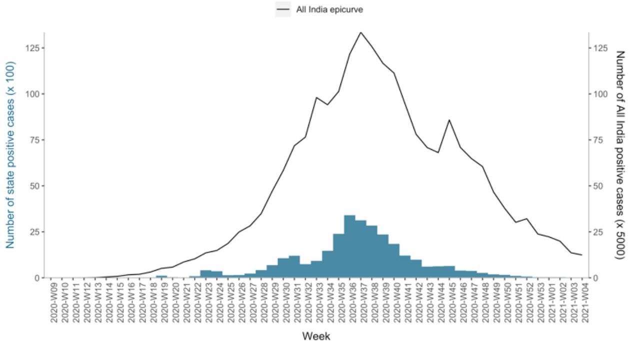

Tripura

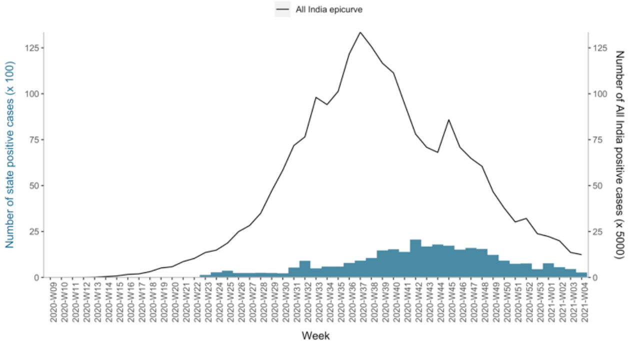

Manipur

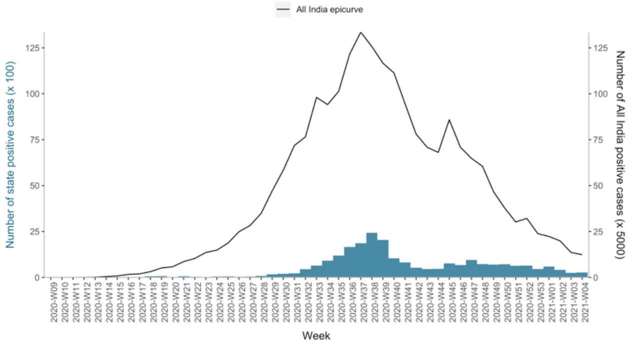

Chandigarh

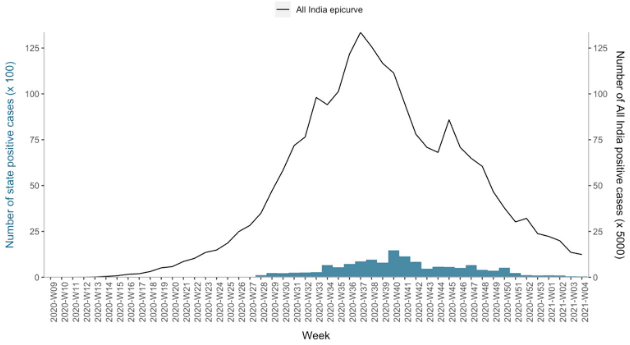

Meghalaya
