# Supplementary material for: COVID-19 testing, timeliness and positivity from ICMR’s laboratory surveillance network in India: Profile of 176 million individuals tested and 188 million tests, March 2020 to January 2021
Source: PLoS One. 2021 Dec 3;16(12):e0260979. doi: 10.1371/journal.pone.0260979 (PMC8641892; doi:10.1371/journal.pone.0260979)
Supplement: Attachment — Submitted filename: PlosOneReview(Saadah).docx [file pone.0260979.s011.docx]

**Abstract**

*Background*: The Indian Council of Medical Research set-up a pan-national laboratory network to

diagnose and monitor Coronavirus disease 2019 (COVID-19). Based on these data, we describe

the epidemiology of the pandemic at national and sub-national levels and the performance of the

laboratory network.

*Methods*: We included surveillance data for individuals tested and number of tests from

March 2020 to January 2021. We calculated incidence of COVID-19 by age, gender, and state

at the individual level and tests per 100,000 population, proportion of symptomatic individuals among those tested, proportion of

repeat tests, and test positivity. We computed median [Interquartile range

(IQR)] days needed for selected surveillance activities to describe timeliness.

*Results*: The analysis included 176 million individuals and 188 million tests. Overall incidence

of COVID-19 was 0.8% and 12,584 persons per 100,000 population were tested. Person

positivity was 6.1%. Ten of the 37 Indian States and Union Territories accounted for about

75% of the total cases. Daily testing scaled up from 40,000 initially to nearly one million in

March 2021. Median duration between symptom onset and sample collection was two

(IQR=0,3) days, while median duration between both sample collection and testing and between testing and

data entry were less than or equal to one day. Missing or invalid entries ranged from 0.01% for

age to 0.7% for test outcome.

*Conclusion*: The laboratory network set-up by ICMR was scaled up massively over a short

period of time, which enabled testing large sections of the population. Although all states and

territories were affected, the majority of cases was concentrated in a few large states. Duration

 between the various surveillance activities was acceptable indicating good responsiveness

 of the surveillance system.

**Introduction**

 The coronavirus disease 2019 (COVID-19) pandemic caused by the novel severe acute respiratory

 syndrome-coronavirus-2 (SARS-CoV-2) virus has presented a monumental challenge to

 the health and socio-economic situation in India and has caused a level of disease burden second

 only to that of the United States.(1) As the second most populous country in the world spread across

 37 states and Union Territories (UTs), the pandemic has impacted India severely. The key public

 health strategy towards reducing the transmission of COVID-19 is testing suspected and high-

 risk individuals followed by tracking and contact tracing. This strategy relies mainly on

 laboratory confirmation of COVID-19 by real-time reverse transcription polymerase chain

 reaction (RT-PCR) among suspected and at-risk groups.

 In order to operationalize the COVID-19 testing strategy, the Indian Ministry of Health and

 Family Welfare established a network of COVID-19 testing laboratories under the auspices of the

 national medical research body, the Indian Council of Medical Research (ICMR). The research

 body was entrusted with the responsibility of framing testing guidelines, setting up a network

 of COVID-19 testing labs, accrediting laboratories for testing, issuing guidelines, and approving

 testing kits. ICMR’s COVID-19 laboratory surveillance network is one of the largest such

 networks established in India for any communicable disease. Initially started in February

 2020 with a small number of national and regional labs, the network has grown to include

 more than 2300 government and private labs across the country as of March 2021. This network

 maintains data on COVID-19 testing performed on every individual in the country.

 Earlier attempts at describing the epidemiology of COVID-19 patients in India has been limited

 to single centre experiences from teaching hospitals.(2–4) Our group had earlier published

 descriptive epidemiological data based on ten million tested individuals.(5) With more than 220

 million samples tested as of March, 2021 and the evolution of the pandemic, analysis of data

 from this network can provide epidemiological profiles for affected individuals and the

 characteristics of the pandemic (6) and guide control measures. Though India is one country, every state within it and every district within the states

 function as independent entities when it comes to health of its population.(7)

 In this context, based on the ICMR COVID-19 laboratory surveillance network, we here describe the

 time, place and distribution of persons tested for COVID-19 and documented quality,

 timeliness, and data quality of the surveillance system.

**Methods**

*Data source*: The reported data of the individuals tested at the laboratories and testing

 centres accredited to the ICMR COVID-19 diagnosis and surveillance network .

*Data reference period*: We analysed data from March 1, 2020 to January 31, 2021.

*Description of the ICMR COVID-19 laboratory surveillance network database:*

 To monitor the evolution and spread of the pandemic in real-time, 2294 laboratories

 (as of January, 2021) and testing centres were required to enter their records via a web

 application designed specifically for this purpose. A standalone system was created to

 manage the data that were being collected. The centralised database was managed at the

 ICMR headquarters in New Delhi. The database was designed with a individual-level table

 containing personal characteristics and a test-level table containing the test characteristics in

 MySQL. All the reporting units in the network entered the data on a daily basis from all the

 districts of the 37 states and union territories of India.

*Data entry process*

 The data of each individual tested for the SARS-CoV-2 infection was entered into the application on

 the day of sample collection. The collected and entered data included demographic details,

 reported symptoms, travel history, patient category, and contact history. Outcome of the RT-

 PCR test was entered as three sperate results, one each for the E, RdRP and Orf genes. Each

 record was updated with the test outcome when the results became available.

*Data management*

 We extracted data from the database within the reference period.

 We linked the individuals tested to their results by means of a unique ID with

 separate unique IDs used for multiple testing of an individual. After extraction, we cleaned the

 data using range checks for dates and continuous variables and legal options for categorical

 variables. The detailed work flow of data cleaning is given in Supplementary figure 1. Due to

 the large size of the data, all calculations were performed in a server and the final tables alone

 exported to the local computer.

*Statistical analysis*

 Analysis was carried out at the individual and test levels by person, time, and place. At the tested

 individual level, number of positive cases by age, gender, State/UT, and ISO (International

 Organization for Standardization) week were calculated. Persons tested and incidence (log

 transformed) per 100,000 population and percentage of individuals who tested positive were

 also calculated in a similar way. Population denominators for states and districts were

 projected for 2020 using the decadal growth rates calculated between the previous two

 censuses.

 We generated epi-curves at the national level and by major states along with weekly percent

 change over ISO weeks. We constructed epidemic curves based the dates of laboratory

 confirmation (as entered in the database), symptom onset, and sample collection. Epi-curves

 were generated for selected states across three burden categories based on total positive

 cases (<100,000, 100,000 to 400,000 and >400,000) and compared with that of epi-curve at

 the national level.

 We created area maps at the administrative (state) level and cumulative

 incidence by ISO week at the district level to depict indicators such as persons

 tested and positive cases per 100,000 population and percentage positive among tested.

 At the tests level, tests per 100,000 population, % symptomatic among tested, % repeat tests,

 and test positivity were calculated by ISO week. Increase in the testing capacity of the

 surveillance network was captured across three scale-up phases (Phase I – March to May

 2020, Phase II – June to August 2020, and Phase III – beyond August 2020). In order to describe

 the timeliness of the system, we calculated time-interval for three categories: symptom onset

 to sample collection, collection to testing, and testing to data entry. We calculated median

 [Inter Quartile Range (IQR)] of these time intervals by scale-up phase, test outcome, and

 location. All analyses were carried out using R server edition version 4.0.3 and QGIS version 3.16.

 Research ethics: Analysis was carried out on de-identified data. Individuals with access to the

 database did not retrieve any data on individual address, phone number, or name. The study

 protocol was approved by the ICMR-COVID-19 National Ethics Committee (CoNEC) of the ICMR.

**Results**

*Testing and Incidence by age, gender and location*

 During the reference period, 176 million individuals and 188 million tests were recorded in

 the surveillance system. Per 100,000 people, the number tested were

 12,583 overall, 14,642 among males, and 10,392 among females. The overall incidence per

 100 population was 0.8, with 0.9 among males and 0.6 among females. The highest incidence

 (1.5 per 100 population) was in the >60 age group compared to 0.2 in the 0-17 and 1.1 in the

 18-60 age groups. About 6.1% of the persons tested were positive for SARS-CoV-2, with the

 highest positivity (10.9%) among those aged above 60 years.(Table 1) The southern and northern

 most states had a higher frequency of persons tested per 100,000 population as compared

 to the central, western and north-eastern regions. Delhi with 47,610 persons tested per

 100,000 population ranked first whereas Uttar Pradesh, Madhya Pradesh, Rajasthan and

 West Bengal had the lowest numbers among the larger Indian States.(Figure 1A,

 Supplementary table 1) Spatial distribution of incidence was similar to that of testing with the

 striking exception of Maharashtra which showed high incidence and positivity (despite

 moderate testing) and Telangana which showed low incidence and positivity (despite higher

 testing). The highest incidence was seen in Goa (3.7%) and Delhi (3.3%) and the highest positivity

 was seen in Maharashtra (15.5%) and Goa (13%).(Figure 1B-C and Supplementary tables 1-2)

 Detailed results can be found at https://sarizwan.shinyapps.io/ICMRCOVID_RIZWAN/.

*Time trends in incidence and epi-curves by age, gender and location*

 Over time, cases increased steadily reaching a peak during week 37 in 2020 and then

 briefly declined, then again increased reaching a second (lower) peak around week 45, before continuing a steady

 prolonged decline.(Figure 2A) Incidence was consistently higher among those aged >60

 and among males throughout the reference period whereas the absolute case load

 was largely attributed to those aged 18-60 years and by males.(Figure 2A-D) Epi-curves by states

 indicated that the early phase of the pandemic was driven predominantly by Maharashtra,

 Andhra Pradesh, Karnataka, Tamil Nadu, Delhi, and Uttar Pradesh, whereas the later part was

 driven by the states of Kerala, Delhi, Rajasthan, West Bengal, Odisha, Haryana, Uttarakhand,

 Goa, Himachal Pradesh, and Manipur.(Figure 3A and Supplementary figure 2) Kerala reported

 the highest weekly percentage increase in terms of absolute case count at ISO week 52 (Figure

 3B), whereas states like Maharashtra, Haryana, Madhya Pradesh, Uttarakhand, Goa, and

 Himachal Pradesh showed a bi-modal distribution with a tri-modal distribution observed

 in Delhi and Gujarat.(Supplementary figure 2)

 Incidence over time and space at the district level documented early spread in the southern

 and coastal states of Kerala, Tamil Nadu, Maharashtra, Karnataka, Orissa, and Ladakh and

 a few districts in the North-East as compared to the central and western states. All the

 districts reported at least one case for the first time during the week ending on 16 August

 2020 (ISO week 33).(Figure 4 and Video figures 1 and 2) Among the southern states,

 Telangana was an outlier as following an initial surge, the cumulative incidence remained fairly consistent.(Figure 4II.B) Even at the height of the pandemic (Figure 4II.A) when

 almost all the districts in the south were experiencing >20 cases per 100,000 individuals per week,

 Telangana reported much fewer cases and even towards end of reference period (Figure

 4III.A), it was the only larger state, wherein half of the districts stopped reporting cases. At

 the end of the reference period (Figure 4III.A) when most parts of India were still reporting

 fewer than 3 cases per week per 100,000 individuals, the whole of Kerala and a few pockets in

 western and north-eastern Maharashtra were reporting >20 cases per 100,000 individuals.

 The epi-curves based confirmation date was shifted to the right as compared to the curve

 based on sample collection date, despite the fact that the peak weeks did not differ between

 these two curves (Figure 5A). However, we noted an unusually high number of cases

 confirmed on week 45, whereas this was not the case with the sample collection curve.

 Examination of the state epi-curves showed that Kerala declared many cases on week 45,

 coinciding with the national curve, which may be due to cumulative reporting of a substantial

 number of cases in week 45.(Supplementary figure 2) The epi-curves for symptomatic

 cases when drawn with dates of symptom onset, sample collection, and confirmation show that the

 symptom onset date curve predated the other two curves by a week.(Figure 5B)

*Performance of the testing*

 Number of tests per 100,000 individuals reached an aggregate of 13,431 at the national level

 during the reference period. Of the tests conducted, 7% were among symptomatic

 individuals, 5.1% were repeat tests, 6% were positive, and 0.7% had no outcomes

 reported.(Table 2) Scale-up of the testing facilities from just a single laboratory in January

 2020 to that of more than 2300 labs in January 2021 was reflected in the steady increase of

 daily tests from 40,278 in phase I and 405,966 in phase II to that of 962,716 tests

 in phase III. (Table 2) However, frequency of daily tests declined slightly towards the end of

 the reference period in January 2021. Excluding the initial high proportions, the trend of test

 positivity increased up to week 31, after which it declined steadily. Similarly, the percentage

 of tests carried out among symptomatic individuals was very high up to week 29 after which

 it declined. Percentage of repeat testing remained relatively constant throughout the

 reference period but declined towards the end coinciding with the decline in overall

 testing.(Figure 6)

 Delhi had the highest testing proportion with 51,414 tests per 100,000 individuals. Telangana

 (24.1%) and Gujarat (18%) performed the most testing among symptomatic persons. The

 state with the highest proportion of repeat testing was Andhra Pradesh (11.8%) followed

 closely by Nagaland (9.1%) and Uttar Pradesh (8.6%). Except for Arunachal Pradesh and

 Lakshadweep, all other states and UTs tested more in phase III than in the earlier

 phases.(Supplementary table 3)

*Timeliness of the surveillance network*

 Median (IQR) duration between symptom onset and sample collection was 2 (Range: 0 to 3)

 days. Results of sample testing were entered on the day of collection itself

 or the next day. There was no difference in the durations between a

 negative and positive test, except for a longer IQR (1 to 4 days) for symptom to collection

 time for positive tests as compared to (IQR=1,3) for negative tests. Across the scale-up phases,

 the symptom to collection duration decreased from 3 days to 2 days and the other intervals

 decreased from 1 day to zero day with their distributions became much tighter.(Table 3, Figure

 7)

 Among the states, symptom to collection duration varied from zero to 3.5 days and collection

 to testing duration was typically less than one day. In contrast, the testing to entry

 time was longer for Andaman and Nicobar Islands (13 days), Kerala (10 days) and Nagaland (4

 days). In general, union territories and north-eastern and island states experienced longer durations

 than those of the mainland states across test results and scale-up phases. Positive tests were

 generally entered more quickly or as quickly as negative tests with the exception of

 Nagaland and Telangana. Nagaland took the longest time (7 days) to enter positive tests

 followed by Kerala (5 days) and Andaman and Nicobar Islands (4 days).(Supplementary tables

 4-6)

**Discussion**

 In the context of the COVID-19 pandemic in India, we reviewed data on 176 million

 individuals tested in 2294 laboratories of the countrywide ICMR COVID-19 surveillance

 network during the period March 2020 to Jan 2021. Through this analysis, we profiled the descriptive

 epidemiology of COVID-19 in India and documented performance-related indicators of a

 large network of laboratory surveillance.

 The cumulative incidence during the reference period was 0.8 per 100 individuals which was

 105th in the world, similar to countries like Malaysia, Saint Vincent and the Grenadines,

 Finland, El Salvador, Guatemala, Botswana and Nepal.(8) Testing per 100,000 individuals in

 India was 13,431 which was 156th in the world and comparable to countries like Morocco,

 South Africa, Iraq, and Malaysia.(9) Test positivity percentage was 6% which was about 50th in

 the world and comparable to countries like Morocco, Kuwait, Togo, Cote d'Ivoire, Qatar,

 Ireland, and France.(10)

 Many known epidemiological facts about the COVID-19 pandemic such as higher incidence

 among males and the elderly was observed in the Indian context.(11,12) The higher incidence

 among elderly was probably due to their aging immunological and physiological status along with the

presence of co-morbidities such as diabetes and hypertension.(13,14) Similarly, the

 higher incidence in males could be attributable to biological (such as higher expression of

 angiotensin-converting enzyme-2 receptors) and sociological (such as poorer lifestyle choices

 and riskier behaviour) factors .(15)

 The pattern of spatial distribution of COVID-19 in India, where certain states like Maharashtra,

 Delhi, Tamil Nadu, Andhra Pradesh, Kerala, and Karnataka were severely affected in the early

 stages of the pandemic could be explained by the high level of urbanisation and population

 density as compared to the other states.(16,17) The distribution in Ladakh was unique since it

 is a tourist destination and the peak in the initial phase could have been influenced by

 imported cases.

 Changes in the testing strategy occurring over the different phases of the pandemic were

 reflected in the upward trends in the frequency of asymptomatic individuals and the

 declining trends in repeat testing.(18) Initially, when the testing strategy was targeted by risk

 category, the percentage of symptomatic individuals tested was high. However, later on

 when the criteria was made more inclusive, the same indicator tapered off to around 7-10%.

 Changes in testing criteria were also reflected in the percentage of repeat testing of the

 individuals. During the early phases, tests were repeated every week or so for admitted

 patients and a negative test was required before discharge. When this criterion was

 relaxed, the repeat testing proportions tapered to around 5%. This repeat testing proportion

 could also be considered an indirect indicator of repeat infections if only symptomatic

 individuals were to be considered.

 As the pandemic progressed, the testing of asymptomatic individuals may reflect the

 performance of the testing strategies. Certain states like Andhra Pradesh, Gujarat, and

 Madhya Pradesh still continued to test a higher proportion of symptomatic individuals despite

 this fact. This shows that states followed different strategies for testing individuals despite

 regular revisions of ICMR testing strategies.(19) The highest proportions of people tested were

 in Delhi, Goa, Andaman, and Nicobar Islands and Puducherry, all of which were union

 territories or very small states with respect to population size. On the other hand, Madhya

 Pradesh, Goa, and Nagaland had the highest positivity proportion as compared to the other

 states. Although higher positivity indicates increased transmission, a detailed investigation on

 why certain states experience exceptionally high positivity needs further investigation. The

 massive scale-up of testing facilities across the Indian sub-continent was captured in great

 detail by our data analysis, from merely 40,000 tests per day in the early phase of the

 pandemic to that of almost 1 million tests per day in 2021. Indeed, between weeks 27 and 39,

 testing numbers increased exponentially. One of the most important strategies in the fight

 against COVID-19 was more testing and more isolation. This strategy was implemented by

 ramping up the testing to hilt capacity within a record time, albeit not uniformly across

 the country. The scale-up reduced the time intervals for sample collection and testing and

 then reporting. The median time taken for testing since onset of symptoms was three days in

 the early phase but reduced to two days in the latter phase. This could be attributed to the

 constant messaging to the population to get tested as soon as they had any symptom.(20)

 Each state in India experienced the pandemic differently. Although the reporting was

 coordinated by the federal Government, the States differed in the rigor of reporting cases to

 the laboratory network. In general, almost all the states and UTs were prompt in entering the

 data. The State of Kerala was notable, since it is one of the mainland states that experienced

 considerable delays in entering positive and negative cases into the database, resulting

 in an anomalous peak in the national epi-curve at around week 45. Another exception was

 of Telangana, where the number of cases reported became static beyond a point with

 many districts reporting zero cases towards the end of the reference period. Such decline

 needs scrutiny regarding the factual versus reporting inadequacies. In fact, Telangana was the

 only southern state that reported an incidence of <1 per 100 individuals. Reasons for such

 variations across states warrant further investigation.

 At the national level, the ICMR COVID-19 laboratory surveillance network provided inputs for

 the national level dashboard for the total number of tests conducted and number of positives

 on a daily basis. However, no disaggregated information was provided. Each state developed

 their own methods of data analysis to steer public health measures of quarantine or

 containment based on positivity or caseload. It worth mentioning that the federal Ministry of

 Health and Family Welfare operated a system of data collection where each state and union

 territory reported daily aggregate counts of active cases, recovered individuals, and deaths.(21)

 Strength and limitations

 The key strength of the analysis is the extensive epidemiological profiling of the COVID-19

 pandemic based on nearly two hundred million individual records being reported to the second

 administrative level in India. We were able to provide insights like the number of symptomatic

 persons tested, number of repeat tests, and the evolution and spread of COVID-19 over time

 and space at the district level for the first time. We could provide measures of the timeliness

 of the surveillance system by depicting the median intervals from symptom onset to sample

 collection to testing to data entry.

 The analysis has few limitations, dictated by the quality of the data available. We did not

 detect all duplicate entries of persons in the database as that would require retrieving

 several identifiers. We did remove duplicate entries of the same tests made by the reporting

 centres to as great an extent as possible. It was not possible to estimate the percentage of

 records that are potential duplicates. However, the numer of duplicates is likely to be

 small and unlikely to change the interpretations or modify the

 trends of the here-presented indicators over time.

 Though different kinds of testing kits (like Rapid Antigen Tests and different brands of RT-PCR)

 with varying diagnostic validity were employed to detect the infection, we were unable to

 analyse the impact of this on the test positivity. A mix of different

 brands of RT-PCR test and RAT were used to confirm the diagnosis. Each of these tests has a

 different sensitivity and specificity with sensitivity being higher or equal to specificity. Given this

 practice, indicators like incidence and test positivity which are dependent on positive results

 are likely to be dependent on the proportions of the different tests used. The influence of the

 test validity and the mix of different tests on such indicators warrants further investigation.

 Missing or invalid data was highest for test outcome (0.7%) and ranged from 0.01% (for

 age) to 0.08% (for date of testing). With regard to the degree of missing information, we

 used case-based deletion where all available information for a particular analysis was used.

 Less than 1% of the data were missing and missing data were less prevalent in

 the later phases of the pandemic. As such, we assume our analysis was not greatly affected by this issue.

 Conclusions

 On the basis of analysis of surveillance data from ICMR COVID-19, we conclude that 1.

 Incidence of COVID-19 was higher among elderly and men, 2. States within India and districts

 within the states experienced the pandemic differently at different points of time, 3. The

 national test positivity stabilized at 6%, with a low proportion of testing among

 symptomatic individuals and of repeated tests, 4. As the surveillance network expanded through the

 scale-up phases, time delays narrowed between collection, testing, and reporting and 5.

 The data management system had certain issues (such as duplicate entries and

 missing data) and data validity was dependent on the individual reporting units and state-

 level authorities.

 Recommendations

 On the basis of conclusions, we recommend real-time use of the ICMR COVID-19 laboratory

 surveillance database to guide public health measures and testing strategies at the state or

 district level. Secondly such large databases may benefit from having internal validity checks

 at the time of data collection, entry, and analysis so as to reduce potential errors. As a long-

 term strategy, a plan for establishing data linkages during public health emergencies across

 databases from laboratory, hospitals, and public health authorities in order to generate real-

 time intelligence at various levels on the burden, status of interventions, and their outcomes.
