# Supplementary material for: COVID-19 testing, timeliness and positivity from ICMR’s laboratory surveillance network in India: Profile of 176 million individuals tested and 188 million tests, March 2020 to January 2021
Source: PLoS One. 2021 Dec 3;16(12):e0260979. doi: 10.1371/journal.pone.0260979 (PMC8641892; doi:10.1371/journal.pone.0260979)
Supplement: Attachment — Submitted filename: Responses to Reviewer_TJ.docx [file pone.0260979.s012.docx]

**Responses to Reviewer's comments**

**Reviewer #1:**

Query 1: The language still needs editing. Especially the re-written/added paragraphs should be checked.

Response: We thank the reviewer for this comment. We have now professionally edited the language to meet the journal standards. The changes made can be seen in the track-change document.

Query 2: It should be clarified in the tables and figures whether the numbers refer to tested individuals or tests.

Response: We thank the reviewer for this comment. We have changed the table and figure titles to clearly indicate individuals and tests as follows.

Table 1. COVID-19 testing, incidence and positivity by gender and age among tested **individuals**, India (March 2020 to January 2021)

Table 2. Characteristics of the COVID-19 **tests** carried out in the laboratory surveillance network, India (March 2020 to January 2021)

Fig 1. State-wise distribution of COVID-19 testing, incidence and positivity among tested **individuals**, India (March 2020 to January 2021)

Fig 2. Distribution of laboratory-confirmed COVID-19 **cases** by week and demographic characteristics, India (March 2020 to January 2021)

Fig 3. Distribution of laboratory-confirmed COVID-19 **cases** and percentage change by week and the state of residence, India (March 2020 to January 2021)

Fig 4. Weekly and cumulative incidence of laboratory-confirmed COVID-19 **cases** by the district of residence, India (March 2020 to January 2021)

Fig 5. Distribution of laboratory-confirmed COVID-19 **cases** by dates of symptom onset, sample collection and result confirmation, India (March 2020 to January 2021)

Fig 6. Trends of the characteristics of the COVID-19 **tests**, India (March 2020 to January 2021)

Table 3. Timeliness of the COVID-19 **tests** carried out in the laboratory surveillance network by scale-up phases and test results, India (March 2020 to January 2021)

Fig 7. Timeliness of the COVID-19 **tests** carried out in the laboratory surveillance network by scale-up phases and test results, India (March 2020 to January 2021)

Query 3: The age group 18-60 could be divided in smaller groups. 18-year old people are very different from 60-year old people. Even though the results would not change it would be reasonable to divide this group into at least two groups 18-40, 41-60.

Response: We thank the reviewer for this comment. We have added the indicators for the suggested age groups in all the relevant tables, figures and description.

**Reviewer #3:**

Query 1: “India’s COVID-19 testing strategy evolved with the changing needs of the pandemic. In March 2020, when the testing resources were limited, only high-risk symptomatic individuals, international inbound travellers, high-risk contacts and patients with Severe Acute Respiratory Illness (SARI) were eligible for testing. During the later phases of the pandemic, the eligibility criteria was expanded to include, asymptomatic contacts, surveillance of symptomatic persons in containments zones, patients with Influenza Like Illness (ILI) and, migrant workers. More recently, patient-initiated testing was also added on where situations such as international travel, screening before undergoing medical/surgical procedures were also eligible for testing. A number of public-centric initiatives were taken by the government to provide free and wide access to testing. Persons who wanted testing were asked to contact the nearest public health care facility where testing was provided free of cost. Testing was carried out at homes of symptomatic patients/contacts of laboratory confirmed cases by healthcare workers during routine house-to-house fever surveillance. Mobile vans located at strategic places in major towns and cities also provided testing facilities to people who wanted to get tested. Apart from these government initiatives, many private labs and hospitals also provided fee-based testing.[2,3]”

It could be relevant to give some specific timings instead of “more recently”. If they varied too much between regions it should be refered.

Response: We thank the reviewer for this comment. The guideline for patient-initiated testing (also known as Test-on-demand) was issued in September, 2020 across the country. We do not have information on where the implementation of this guideline was uniform or not. The indicated line has been modified as given below.[Line 101]

“Since September 2020, patient-initiated testing was also added to the criteria, along with situations such as international travel, screening before undergoing medical/surgical procedures were also eligible for testing.”

Query 2: Are there any other reasons (beyond broader testing) why 90% of tested people were not symptomatic? Could there be information bias (social desirability?) people may not report symptoms to the health professional who is collecting the data and testing?

Response: The percentage of tests that were carried out among symptomatic individuals was 7%. We attribute this mainly to the broad nature of the testing criteria used (including contact tracing, international travellers, migrant workers and health care workers). We are unable to comment on whether a social desirability bias was operating where patients were unwilling to disclose to symptoms. Having said that there is no evidence in literature from India to suggest that individuals would refrain from disclosing their symptoms at the time of testing. Also, the symptom information was collected at the time of testing and the individuals could have developed symptoms during their course of illness.

Query 3: “It is likely that the laboratory surveillance system suffered from a certain degree of under-ascertainment of cases. This could be attributed to various reasons. Firstly, testing was voluntary and done only among those who satisfied the eligibility criteria. Since >90% of the infections were asymptomatic, this testing strategy was likely to have missed them and secondly, the sensitivity of the testing kits ranged from 70 to 90%, which again would have led to misclassifying cases into false negatives. Evidence for this under-ascertainment comes from the infection-to-case ratios (ICR) estimated over time from the three rounds of nation-wide sero-surveys in India [20,41,42], which estimates the number likely infections undetected in the community for every case identified by the surveillance. However, the ICR decreased from 81.6 (in May, 2020) to 26 (in August, 2020) to 27 (in December, 2020) suggesting that the sensitivity of the surveillance improved over time.”

It is not clear that “Since >90% of the infections were asymptomatic, this testing strategy was likely to have missed them”. Is this correct? What doess it means. I though 90% of those tested were asymptomatic. If 90% of infections were asymptomatic this warrants further discussion as this is very different from what is found in many other countries.

Response: We thank the reviewer for pointing this out.

In general, the actual percentage of asymptomatic COVID-19 infection varies. According to a systematic review [https://www.pnas.org/content/118/34/e2109229118], 42.8% of COVID-19 affected individuals were asymptomatic at the time of testing and 35.1% were truly asymptomatic, in that they did not develop any symptom throughout the course of the infection. So, a significant proportion of the those COVID-19 infections were asymptomatic. In India also, studies [https://www.ijidonline.com/article/S1201-9712(21)00442-2/fulltext#secsect0085] have reported that asymptomatic COVID-19 infection was very prevalent and this is also affected by the emergence of new variants like Delta which have a lower proportion of asymptomatic infection. The general point that we are trying to make is that the surveillance system may have missed these asymptomatic infections. To reflect this more accurately in the manuscript, the sentence has been rephrased as follows.[Line 787]

“Since a significant proportion of the COVID-19 infections were likely asymptomatic, this testing strategy may have missed them, and secondly, the varying sensitivity of the testing kits would have led to misclassifying cases into false negatives.”

Query 4: “Evidence for this under-ascertainment comes from the infection-to-case ratios (ICR) estimated over time from the three rounds of nation-wide sero-surveys in India [20,41,42], which estimates the number likely infections undetected in the community for every case identified by the surveillance. However, the ICR decreased from 81.6 (in May, 2020) to 26 (in August, 2020) to 27 (in December, 2020) suggesting that the sensitivity of the surveillance improved over time.” – This is good information. Thank you.

Response: We thank the reviewer for this comment.

Query 5: “Persons who wanted testing were asked to contact the nearest public health care facility where testing was provided free of cost.” - Only if they meet the other criteria correct?

Response: Yes, patients were tested as per the testing criteria.

Query 6: I understand it may be difficult but could you refer how many public health care facilities exist in the country or the average population and dimension of the area it serves? This is not absolutely necessary of course but is relevant for context of accessibility to testing.

Response: We thank the reviewer for this comment. It would not be possible within the scope of this paper to provide an assessment of the number of health facilities in a country as large as India, especially given the diverse nature of the prevailing health care delivery system. However, we have given details of the number of COVID-19 testing laboratories in each state in Table S2. By no means this can be taken a proxy for access to testing because these laboratories were linked to several health facilities who provided the samples collected from the patients.

Query 7: “Further, it was seen that the testing rate was higher in men, than in women across all states, in contrast to developed countries in the west.[22] Among those who were tested, the proportion of women was highest in Andhra Pradesh (46.4%) and lowest in Dadra and Nagar Haveli (23.4%). It has been previously shown that young women in certain states of India were less likely than men to practice key COVID-19 preventive behaviours.[23] Thus, such low frequency of testing behaviour among women was reflected in low prevalence among women as well.” Consider changing “developed countries in the wes”to “high-income countries in the west”

Response: The sentences has been modified as follows.[Line 539]

“Further, it was seen that the testing rate was higher in men, than in women across all states, in contrast to high-income countries in the west.[22]”

Query 8: “The overall percentage of symptomatic individual who were tested was lower in our study. This is due to two main reasons – firstly, the testing criteria was kept relatively broad and a large proportion of testing was carried out among asymptomatic individuals who were contacts of cases or picked up for random testing in containment areas [14,15] and secondly, data on symptoms may have been inaccurately reported, collected, or entered in the database.” This is ok.

Response: We thank the reviewer for this comment.

Query 9: “An important indicator of the efficiency of a nationwide pandemic surveillance system is the timeliness of sample collection after an individual develops symptoms.[37,38] In our analysis, we found that in the initial phases of the pandemic the duration between symptom onset and sample collection was three days. In the subsequent phases, we found that the median duration was reduced to zero. This is an important finding because it means that the surveillance system became more responsive and individuals were becoming increasingly aware of the need to get tested immediately after they develop symptoms. An direct effect of this reduction in delay would be more effective isolation of symptomatic patients, early detection of possible contacts and their quarantine. This could have played an pivotal role in halting the progress of continued viral transmission [39], preventing the progress of infection to severe disease and reduction of complications and mortality.”

-The fact that In the subsequent phases, you found that the median duration from symptom onset to testing was reduced to zero needs to be discussed considering social desirability bias. It seems impossible that everyone is test in the exact day they begin symptoms. This may be a very relevant social phenomnenon for the future of the pandemic globally.

Response: Not everyone got tested on the same day of symptom onset. This value only represents the median value, which means that at least 50% of the individuals got tested on the day of symptom onset. A large proportion of symptomatic patients still did not get tested on the same day. There is no evidence in Indian literature to suggest that individuals would not disclose their symptoms while testing due to social desirability bias. Therefore, we would be unable to comment on this hypothesis. We have added the following sentence to reflect this.[Line 677]

“In subsequent phases, at least 50% of individuals got tested at symptoms onset, with another 25% delaying their testing by 3 to 4 days. This interval is important because it indicates that the surveillance system became more responsive with time, and individuals became increasingly aware that they needed testing immediately after developing symptoms.”

Query 10: “However, other explanations include the possibility that high levels of under-ascertainment of cases could also be operating within the states.”

- This is likely. It should not seem as a remote possibility. Consider rephrasing.

Response: The sentence has been rephrased as below.[Line 565]

“However, the possibility of high levels of under-ascertainment of cases within the states cannot be overstated.”

Query 11: “Although higher positivity indicates increased transmission, a detailed investigation on why certain states experience exceptionally high positivity needs further investigation, including the possibility of under-ascertainment of cases.”
Conside writing “the likely higher under-ascertainement of cases and its implications”.

Response: The rephrased sentence now reads:[Line 622]

“Although higher positivity indicates increased transmission, a detailed investigation on why certain states experience exceptionally high positivity is needed focussing on the high under-ascertainment of cases and its implications.”

Query 12: “Ensuring a robust and sensitive surveillance system that can reduce the level of under-ascertainment of cases is absolutely essential to control transmission, better understand transmission dynamics, and changes in vaccine effectiveness and early detection on new variants of concern that may impact the future course of the pandemic”
This is very relevant. Thanks to the authors.

Response: We thank the reviewer for this comment.
